# Contact-Mediated Inhibition Between Oligodendrocyte Progenitor Cells and Motor Exit Point Glia Establishes the Spinal Cord Transition Zone

**DOI:** 10.1371/journal.pbio.1001961

**Published:** 2014-09-30

**Authors:** Cody J. Smith, Angela D. Morris, Taylor G. Welsh, Sarah Kucenas

**Affiliations:** Department of Biology, University of Virginia, Charlottesville, Virginia, United States of America; Stanford University School of Medicine, United States of America

## Abstract

Rapid conduction of action potentials along motor axons requires that oligodendrocytes and Schwann cells myelinate distinct central and peripheral nervous system (CNS and PNS) domains along the same axon. Despite the importance of this arrangement for nervous system function, the mechanisms that establish and maintain this precise glial segregation at the motor exit point (MEP) transition zone are unknown. Using *in vivo* time-lapse imaging in zebrafish, we observed that prior to myelination, oligodendrocyte progenitor cells (OPCs) extend processes into the periphery via the MEP and immediately upon contact with spinal motor root glia retract back into the spinal cord. Characterization of the peripheral cell responsible for repelling OPC processes revealed that it was a novel, CNS-derived population of glia we propose calling MEP glia. Ablation of MEP glia resulted in the absence of myelinating glia along spinal motor root axons and an immediate breach of the MEP by OPCs. Taken together, our results identify a novel population of CNS-derived peripheral glia located at the MEP that selectively restrict the migration of OPCs into the periphery via contact-mediated inhibition.

## Abstract 2

In vivo experiments in zebrafish determine that CNS-derived glial cells contribute to the myelinating population of cells in the PNS and ensure that CNS and PNS glia remain segregated.

## Author Summary

The nervous system is often thought as two distinct halves: the central nervous system (CNS), which consists of the brain and spinal cord, and the peripheral nervous system (PNS), which includes the nerves that control movement and sense the environment. The cells within these two halves, however, do not commonly mix. To address how cells are segregated within these two compartments of the nervous system, we used live, transgenic zebrafish embryos to watch nerve development. Our study shows that CNS-residing myelinating glia (nonneuronal cells that wrap around nerves to ensure nerve impulse conduction) are restricted from entering the PNS by a cell we call motor exit point (MEP) glia. MEP glia originate from within the CNS, and then migrate into the PNS, divide, and produce cells that ensheath and myelinate spinal motor root axons. Ablation of MEP glia causes CNS glia to migrate inappropriately into the PNS, disrupting the normal boundary that is present between the CNS and PNS. Overall, the identification and characterization of MEP glia identifies an aspect of peripheral nerve composition that may be pertinent in human health and disease.

## Introduction

Traditionally, the CNS and PNS have been thought of as two, distinct halves of one organ system that are fused into a functional unit by bundles of motor and sensory axons. Where these axons cross between the CNS and PNS are known as transition zones (TZs). These specialized structures are recognized by glia, such that oligodendrocytes and Schwann cells, the myelinating glia of the CNS and PNS, respectively, stay segregated at these locations [1]–[4]. However, recent studies have demonstrated that at least some components of the PNS originate from precursors within the spinal cord and can freely pass through these TZs [5],[6]. These data, taken together with the descriptions of ectopic glial populations in both the CNS and PNS when myelin is disrupted [7]–[12], led us to hypothesize that there are normally mechanisms in place that selectively monitor the glial boundary between the spinal cord and periphery and are essential for specifically maintaining the strict segregation of myelinating glia observed at these locations.

In mammals, neural crest-derived boundary cap cells (BCCs) reside at the junction between the CNS and PNS at motor exit points (MEPs) and have been shown to restrict motor neurons from migrating into the PNS [9],[11],[13],[14]. However, their role in glial restriction is less understood as oligodendrocytes and astrocytes have been described in the PNS in both their presence and absence, suggesting that these cells may not be the only population responsible for restricting glial migration into the periphery [7],[10],[14]. Consistent with this, electron microscopy studies have described the cell populations at the MEP TZ as morphologically distinct from those at the dorsal root (sensory) TZ [1],[15]. Furthermore, elegant neural crest ablation studies in chick have demonstrated that even in the absence of neural crest and all of its derivatives, including BCCs, a population of glial cells is still found along spinal motor nerve roots, demonstrating that they originate from a nonneural crest progenitor [16]–[19]. All of these studies led us to hypothesize that there may be a second glial population associated with spinal motor root axons that is distinct from neural crest-derived BCCs/glia and that it is this population that is responsible for segregating myelinating glia at the MEP.

With the goal of determining how myelinating glial segregation is achieved at the MEP during development, we used live imaging in zebrafish to visualize the development of this boundary. Prior to the onset of myelination, we observed oligodendrocyte progenitor cells (OPCs) extend membrane processes into the periphery via the MEP. Immediately upon contact with *sox10^+^* glia along spinal motor root axons, these processes retracted back into the spinal cord. Characterization of the cells that OPC processes contacted during these sampling events revealed that they were distinct from neural crest-derived glia, as they originated within the spinal cord and developed normally even in the absence of neural crest. These CNS-derived cells expressed *sox10*, *olig2*, *foxd3*, and *wif1* and were distinct from previously described perineurial glia, Schwann cells, OPCs, and BCCs. Specific ablation of these CNS-derived peripheral glia not only eliminated all peripheral myelinating glia along spinal motor roots, but also disrupted the MEP TZ, leading to the ectopic exit of OPCs from the spinal cord. From these data, we conclude that CNS-derived peripheral glia are responsible for restricting OPCs to the spinal cord via contact-mediated inhibition. Together, our studies (1) identify a novel population of CNS-derived spinal motor nerve-associated myelinating glia that we propose calling MEP glia and (2) introduce the phenomenon of contact-mediated inhibition between glia across TZs as a mechanism by which the strict segregation of myelinating glia is achieved during development.

## Results

### OPC Membrane Processes Sample the PNS Prior to Differentiation

**Figure 1:**
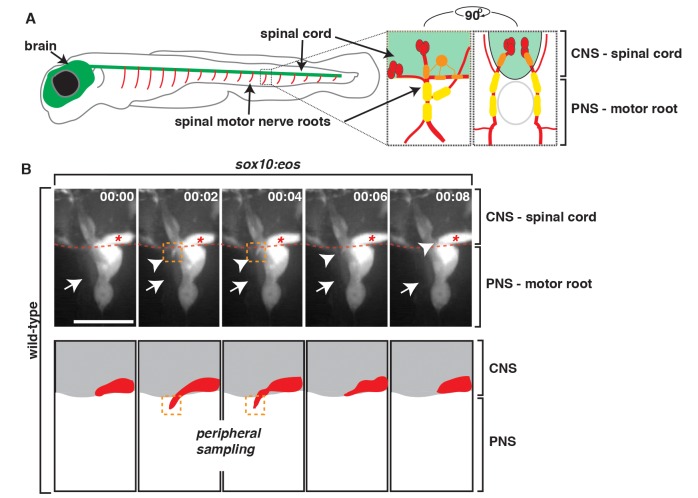
PNS glia restrict OPC membrane processes in the PNS. (A) Schematic of zebrafish spinal motor roots showing the CNS (green) consisting of the spinal cord and brain and the PNS (red). Insets show zoomed representations of a single spinal motor nerve root with motor axons (red), OPCs (orange), and PNS glia (yellow). Lateral and cross-section views are shown. (B) Frames captured from a 14-h time-lapse video beginning at 58 hpf in a *Tg(sox10:eos)* embryo. Numbers in upper right corners denote time lapsed from the first frame of the figure. (A) At approximately 68 hpf (00:00), peripheral glial cells (arrow) and OPCs (asterisk) can be seen. Just 2 min later (00:02), an OPC process (arrowhead/orange box) extended out of the spinal cord, contacted the motor root glial cell (arrow), and quickly retracted, all within 6 min. Traced schematic below shows spinal cord (grey) and OPC (red), at the corresponding times. All images are lateral views of the motor and sensory root with dorsal to the top and anterior to the left. Scale bar, 15 µm.

The mechanisms that mediate how myelinating glial cells are segregated at the MEP TZ are poorly understood. One possibility is that glial cells on either side of the MEP physically interact during development to establish the tight interdigitated glial boundary that has previously been described [1],[20]. To test this hypothesis, we sought to investigate if OPC membrane processes contacted peripheral glia during development. In zebrafish, OPCs associate with the central segment of motor axons located inside the spinal cord, whereas peripheral glia are located along the peripheral portion of motor axons after they extend out of the spinal cord (Figure 1A). Using time-lapse imaging in 60 h postfertilization (hpf) wild-type *Tg(sox10:eos)* embryos, which have *sox10* regulatory sequences driving Eos in both central and peripheral glial cell lineages, we observed short (∼9 µm) *sox10^+^* OPC-membrane processes extend out of the spinal cord via the MEP (Figure 1B and Video S1). The OPC membrane processes were very transient, and we typically observed them in the periphery for no longer than 10 min (Figure 1B). Orthogonal images (yz plane) of these data confirmed that OPC processes physically contacted peripheral spinal motor root *sox10^+^* glia and after contact quickly retracted back into the spinal cord (Figure S1). Between 54 and 72 hpf, we typically observed OPC peripheral sampling one to two times per nerve (*n* = 8 nerves). However, after this stage, OPCs began to myelinate spinal cord axons, and we rarely observed membrane processes in the periphery later than 72 hpf (Figure 1 and Video S1). These data introduce the phenomenon of OPC peripheral sampling and contact-mediated inhibition with peripheral glia, which has previously only been observed between two OPCs within the CNS [21],[22]. Therefore, we hypothesize that this interaction is fundamental to restricting OPCs to the spinal cord at the MEP TZ.

### Nonneural Crest-Derived Glia Populate Spinal Motor Root Axons

**Figure 2:**
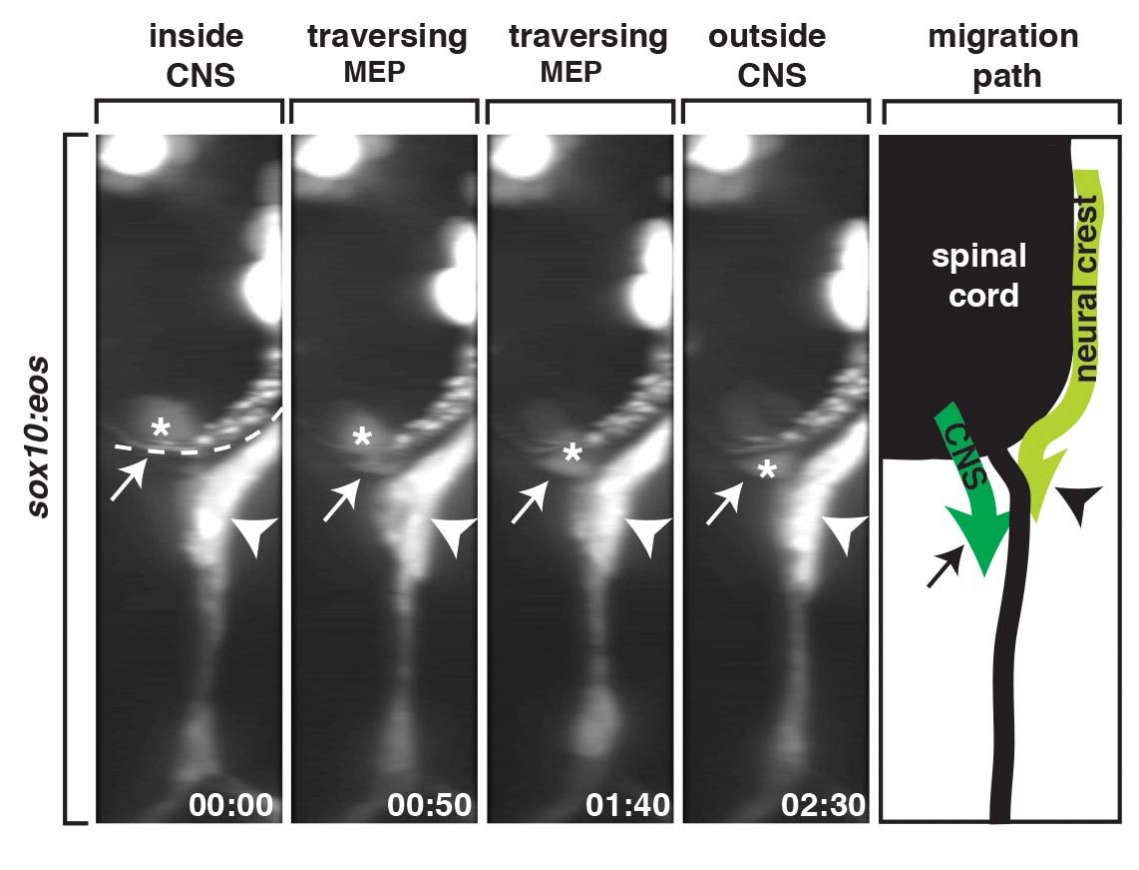
*sox10^+^* motor root glial cells originate in the CNS. A 90-degree rotation of images captured from a 24-h time-lapse video beginning at 48 hpf in a *Tg(sox10:eos)* embryo. Numbers in lower right corners denote time lapsed from the first frame of the figure. At approximately 56 hpf (00:00), a *sox10^+^* cell (asterisk) migrated from the spinal cord, pinched through the MEP (arrow), and migrated into the periphery. Migration path shows the migration of neural crest versus CNS-derived *sox10^+^* cells during this developmental stage. Dashed line marks lateral edge of the spinal cord. Scale bar, 25 µm.

As a first step towards identifying the cells that appear to repel peripherally sampling OPC membrane processes at the MEP TZ, we used *in vivo* time-lapse imaging to carefully define the glial populations at this boundary. Previous reports in both zebrafish and mouse describe the ventral migration of *sox10^+^* neural crest cells along the lateral edge of the spinal cord during early development [23]–[26]. When we continued imaging between 48 and 72 hpf, after neural crest migration had ceased [26], we observed a cell in a distinct, more dorsal and medial location from the neural crest initiate expression of *sox10* (Eos) at 56 hpf, change its morphology to squeeze through the MEP TZ, and migrate ventrally along spinal motor root axons (Figure S2). When we first visualized this cell, it appeared to be in a position that was consistent with it being inside the spinal cord (Figure S2). To better determine if this *sox10^+^* cell originated within the spinal cord, we imaged developing motor nerves in laterally mounted *Tg(sox10:eos)* embryos from 48 to 72 hpf and then digitally rotated the images 90 degrees to view the data in transverse cross-section (Figure 2). Viewing the data in this manner allowed us to see a *sox10^+^* cell located within the ventral spinal cord migrate ventrally towards the MEP, where it altered its morphology to pinch through the TZ (Figure 2 and Video S2). This morphological change is a behavior we previously observed by perineurial glia and is consistent with the hypothesis that they are exiting the spinal cord [6],[27]. Between 48 and 72 hpf, we observed *sox10^+^* cells exiting the spinal cord in this manner at 58% of the MEP TZs we imaged. From these data, we hypothesize that after neural crest migration ceases, *sox10^+^* cells within the spinal cord exit the CNS at the MEP and sit immediately adjacent to *sox10^+^* neural crest cells along spinal motor root axons.

**Figure 3:**
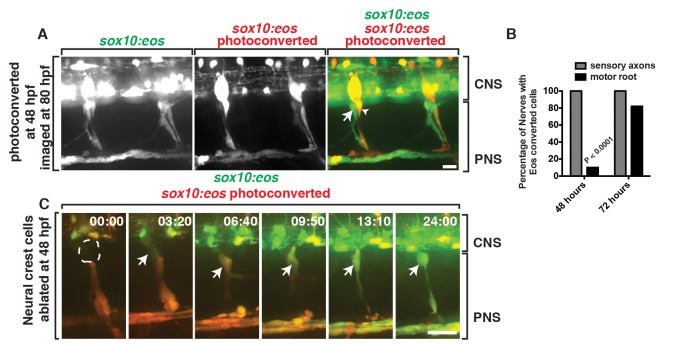
Motor root-associated glia are distinct from neural crest-derived glia. (A) In a *Tg(sox10:eos)* embryo exposed to UV light at 48 hpf and imaged at 80 hpf, photoconverted cells (red, arrowhead) are only observed along sensory axons while unconverted cells (green, arrow) populate the motor root. CNS and PNS portions are denoted in brackets. (B) Quantification of photoconversion experiments shows CNS-derived, unconverted *sox10^+^* cells develop between 48 and 72 hpf, whereas converted *sox10^+^* cells that generate the DRG are present before 48 hpf (30 nerves scored). (C) Frames captured from a 24-h time-lapse video beginning at 48 hpf in a *Tg(sox10:eos)* embryo that was exposed to UV light at 48 hpf and DRG ablation immediately after. Numbers in upper right corners denote stage of development. At 51.3 hpf (03:20), a CNS-derived *sox10^+^* cell (green, arrow) associated with motor axons and generated motor root glial cells even in the presence of dying DRG cells (red). All images are lateral views of the motor and sensory root with dorsal to the top and anterior to the left. CNS and PNS portions are denoted in brackets. Scale bars, 25 µm.

To determine if the *sox10^+^* cells we observed exiting the spinal cord at the MEP were distinct from previously described neural crest-derived glia and not simply misrouted neural crest cells, we used fate-mapping and neural crest ablation. Using *Tg(sox10:eos)* embryos for fate-mapping [26], where the mature Eos protein, when exposed to ultraviolet (UV) light, shifts its emission from a green fluorescent state (516 nm) to a red fluorescent state (581 nm) [28], we first exposed the entire animal to UV light at 48 hpf, when neural crest cells were present along the motor nerve but the later born *sox10* ^+^ cells were not yet visible (Figure S3A). Interestingly, at approximately 56 hpf, we observed a *sox10^+^* cell (green) at the MEP (Video S3). This cell divided and generated unconverted *sox10^+^* cells (green) that associated only with motor root axons, and by 72 hpf, nonneural crest-derived *sox10^+^* cells (green) were loosely ensheathed around both the dorsal and ventral motor root projections (Figure 3A and Video S3). Ultimately, by 80 hpf, both motor and sensory axons were ensheathed by *sox10^+^* cells, with developing sensory root axons populated only by previously photoconverted, neural crest-derived *sox10^+^* (yellow) cells and motor roots ensheathed solely by unconverted, nonneural crest-derived *sox10^+^* (green) cells (Figure 3A,B and Video S3). We confirmed this spatial and temporal segregation of *sox10^+^* cells along motor versus sensory roots at the MEP using two independent *sox10* transgenic lines, *Tg(sox10:megfp)* and *Tg(sox10:mrfp*) [21],[29], in combination with the *Tg(neurod:gfp)* [30] and *Tg(olig2:dsred2)* [6] transgenes, which label sensory and motor axons, respectively (Figure S4). From these data, we conclude that *sox10^+^* neural crest cells first associate with outgrowing motor axons and then reposition posterior to the spinal motor root, giving rise to the DRG and its associated glia during early development. All spinal motor root-associated *sox10^+^* glial cells then arise from a distinct, later-born, nonneural crest-derived *sox10^+^* progenitor that we hypothesize arises from a precursor in the spinal cord.

As an independent confirmation that the cells we observed exiting the spinal cord at the MEP were not neural crest cells, we used laser ablation to remove neural crest cells after their migration had ceased, at 48 hpf, along individual developing spinal motor nerve roots in *Tg(sox10:eos)* embryos that were exposed to UV light immediately prior to ablation. When neural crest-derived cells closest to the MEP were ablated, we observed a *sox10^+^* cell initiate expression in the spinal cord and migrate ventrally out of the spinal cord and associate with a spinal motor root axon (Figure 3C). Interestingly, in these embryos, the DRG never developed, supporting the hypothesis that the neural crest population and the spinal cord population of peripheral *sox10^+^* glia are distinct and cannot compensate for each other. These results are consistent with the hypothesis that the *sox10^+^* glial cell at the MEP is not neural crest-derived.

### Spinal Motor Root-Associated Glia Originate from *olig2^+^* Precursors in the CNS

**Figure 4:**
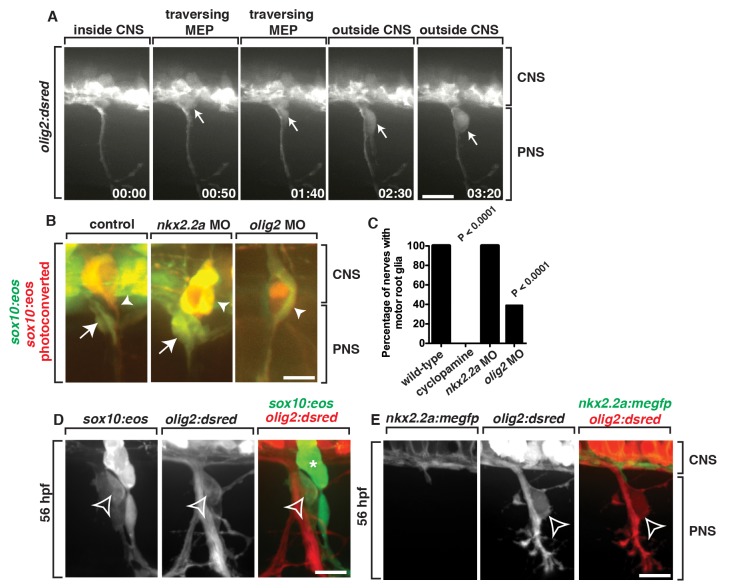
*sox10^+^* motor root glia arise from *olig2^+^* precursors in the spinal cord. (A) Frames captured from a 24-h time-lapse video of a *Tg(olig2:dsred)* larvae showing *olig2^+^* cells (arrow) migrating out of the CNS, pinching at the MEP, and associating with the spinal motor root axons. Numbers in lower right corners denote time lapsed from the first frame of the figure. Brackets show the CNS and PNS portions in the images. (B) In *Tg(sox10:eos)* embryos injected with a *nkx2.2a* MO, CNS-derived *sox10^+^* motor glial cells (arrow) are indistinguishable from wild-type nerves. In contrast, in *olig2* MO-injected embryos, motor root glial cells are absent. In both instances, sensory glial cells are unaffected (arrowheads). (C) Quantification of data from panel B (>50 nerves were scored for each perturbation). (D) In a *Tg(sox10:eos);Tg(olig2:dsred)* embryo at 56 hpf, a *sox10^+^* cell located on the motor nerve root expresses *olig2* and *sox10* (open arrowhead). Neighboring *sox10^+^* cells in the DRG (asterisk) do not express *olig2*. (E) In a *Tg(nkx2.2a:megfp);Tg(olig2:dsred)* embryo at 72 hpf, *nkx2.2^+^* perineurial glia are distinct from *olig2^+^* MEP glia (open arrowhead). All images are lateral views of the motor and sensory root with dorsal to the top and anterior to the left. Scale bars, 25 µm.

We next reasoned that if *sox10^+^* glia along the motor root were derived from the spinal cord, they would express spinal cord-specific markers. During the course of our imaging, we noticed that the cell that initiates *sox10* expression is located in the ventral spinal cord near the MEP (Figure 2). In this region of the spinal cord, precursors in the gliogenic pMN domain express *olig2* and give rise to motor neurons, interneurons, and OPCs [21],[31]–[33]. Using *Tg(olig2:dsred)* embryos, which have *olig2* regulatory sequences driving expression of DsRed in pMN precursors and their descendants [6], we time-lapse imaged between 48 and 72 hpf. In these studies, we observed *olig2^+^* cells exit the spinal cord at the MEP, divide, and remain associated with spinal motor root axons (Figure 4A and Video S4). These data are consistent with our hypothesis that *sox10^+^* spinal motor root glia are CNS-derived and confirm our previous data that demonstrate that they are not misrouted neural crest cells.

To independently confirm that the *sox10^+^* cells we describe originate from precursors in the CNS, we disrupted the development of specific ventral spinal cord domains and scored the presence of *sox10^+^* glia along motor root axons by labeling *sox10^+^* motor root glia with the photoconversion technique described above (Figure S3A). There are two gliogenic precursor domains in the ventral spinal cord: (1) the p3 domain, which expresses *nkx2.2a* and gives rise to CNS-derived perineurial glia and V3 interneurons [21],[32], and (2) the pMN domain [31],[33]. To inhibit a cascade essential for specification of both the p3 and pMN domains, we used cyclopamine (CA), a potent sonic hedgehog (Shh) signaling antagonist [6],[34]. In contrast to control embryos, embryos treated with CA from 8 hpf and imaged at 72 hpf had no motor-associated *sox10^+^* cells, consistent with the hypothesis that spinal motor root glia originate within the ventral spinal cord (Figure 4C). However, a caveat to these studies is that antagonizing Shh affects all cellular components associated with spinal motor nerves, including motor neurons and their associated glia. Therefore, to more specifically perturb individual precursor domains, we injected morpholino oligonucleotides (MOs) into single-cell embryos designed to block translation of either *nkx2.2a* or *olig2* [6],[31],[35]. In *nkx2.2a* MO-injected embryos, we observed *sox10^+^* cells along motor axons at 72 hpf in a pattern indistinguishable from control embryos (Figure 4B,C). These data are consistent with the hypothesis that the *sox10^+^* cells we observed exiting the spinal cord at the MEP were not perineurial glia because they do not require *nkx2.2a* for their development [6]. In contrast, at 72 hpf in *olig2* MO-injected embryos, motor root-associated *sox10^+^* cells were absent from 61% of nerves (Figure 4B,C). Taken together, these data are consistent with our previous imaging data and support the hypothesis that *sox10^+^/olig2^+^* cells originate from *olig2^+^* precursors in the ventral spinal cord and are distinct from *nkx2.2a^+^* perineurial glia (Figure 4D,E).

### CNS-Derived MEP Glia Are a Novel Population of Glia at the MEP TZ

**Figure 5:**
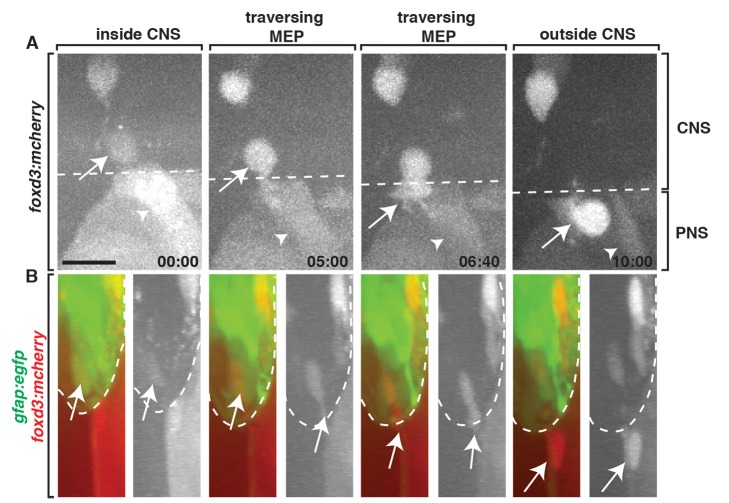
MEP glia express *foxd3*. Frames captured from a 24-h time-lapse video of a *Gt(foxd3:mcherry)* transgenic embryo showed a *foxd3^+^* cell (arrow) located inside the spinal cord (dotted line), migrate ventrally, exit the spinal cord at the MEP, and associate with a spinal motor root axon. The neural crest-derived population (arrowhead) is posterior to the CNS-derived cell. (B) Frames captured from the above video of a *Tg(gfap:egfp);Gt(foxd3:mcherry)* embryo rotated 90 degrees showed a *foxd3^+^* cell starts inside the spinal cord, as marked by *gfap^+^* endfeet along the lateral edge of the cord, then migrates through the TZ, and associates with spinal root axons in the PNS. Scale bar, 25 µm.

Our data demonstrate that the CNS-derived *sox10^+^* glia we observe along spinal motor root axons are distinct from previously described CNS-derived perineurial glia and neural crest-derived cells (Figures 3 and 4). To rule out the possibility that they are OPCs that have ectopically exited the spinal cord, we assayed whether the cells that exited the spinal cord express *foxd3*, a marker that is not expressed by OPCs but is expressed by all known *sox10^+^* peripheral glia [36]. Using *Gt(foxd3:mcherry)* embryos, which have the coding sequence for mCherry inserted into the endogenous *foxd3* locus and have been reported to exactly mimic endogenous *foxd3* expression, which includes expression in the ventral spinal cord [36], we imaged *Gt(foxd3:mcherry);Tg(gfap:egfp)* embryos from 48 to 72 hpf. In these embryos, *gfap* regulatory sequences label radial glial cells and their endfeet, which are located along the lateral edge of the spinal cord and serve as a landmark for the boundary between the CNS and PNS (Figure 5) [37]. At approximately 56 hpf, *foxd3^+^* cells in the ventral spinal cord migrated towards the MEP, squeezed through the TZ, and continued to migrate into the periphery along spinal motor root axons (Figure 5 and Video S5). While in the spinal cord, these *foxd3^+^* cells did not display the highly branched filopodial-like morphology that is typical of OPCs (Figure 5) [21]. Based on these molecular and morphological findings, we conclude that the glial cells we observe exiting the CNS originate from the spinal cord as a novel, uncharacterized glial progenitor that expresses *foxd3*, *sox10*, and *olig2*. Therefore, we propose calling them MEP glia to denote their location and distinguish them from neighboring glial populations.

### Development of MEP Glia Is Dependent on Early Ventral Spinal Cord Precursors

**Figure 6:**
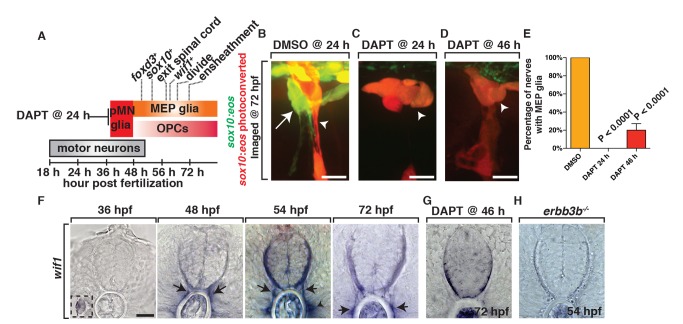
MEP glia originate from ventral spinal cord progenitors and express *wif1*. (A) Summary of MEP glial development and the timing of marker expression. *olig2* is not included because the initiation of expression is difficult to determine with surrounding *olig2^+^* cells already present during this stage of development. (B–D) In *Tg(sox10:eos)* embryos exposed to UV light at 48 hpf and imaged at 72 hpf, (B) DMSO-treated animals have MEP glia, whereas (C) embryos treated with DAPT at 24 hpf and (D) 46 hpf do not. (E) Quantification of data from panel D. (F) *In situ* hybridization with *wif1* riboprobe at 36, 48, 54, and 72 hpf shows timing of *wif1* expression at the root denoted by arrows. Arrowhead denotes the horizontal myoseptum staining. Inset at 36 hpf shows *wif1^+^* staining is expressed at the lateral line, as has been previously described. (G–H) At 54 hpf in *erbb3b* (H) mutants and at 72 hpf in embryos treated with DAPT at 46 hpf (G), we observed an absence of *wif1* staining along spinal motor roots. Scale bars, 25 µm.

To begin to investigate the development of MEP glia, we first determined the developmental timing of *foxd3*, *sox10*, and *olig2* expression using transgenic embryos that expressed [*Gt(foxd3:mcherry)*, *Tg(olig2:dsred)*, and *Tg(sox10:eos)*]. This analysis revealed that MEP glia express *olig2* before they exit the spinal cord and continue to show DsRed fluorescence until approximately 72 hpf (Figures 4 and 6). mCherry expression from the *Gt(foxd3:mcherry)* transgene was observed as early as 46 hpf before they migrated into the periphery and continued until 8 d postfertilization (dpf) when we concluded our imaging (Figure 6A). Lastly, we detected *sox10* expression in MEP glia after they initiated expression of both *olig2* and *foxd3*, at approximately 50 hpf, shortly before they exited the spinal cord. However, this expression was transient, as we only detected *eos* expression out until approximately 8 dpf (Figure 6A).

To gain further insight into MEP glial specification, we investigated if they were generated from the same early ventral spinal cord precursors that generate OPCs. Previous studies have demonstrated that the pMN glial precursors that produce OPCs are dependent on Notch signaling [37]. To test if MEP glia are specified from Notch-dependent pMN precursors, we treated *Tg(sox10:eos)* animals with DAPT at 24 hpf and used the photoconversion paradigm described above at 48 hpf to differentially label MEP glia (Figure S3A). When we imaged these DAPT-treated larvae at 72 hpf, MEP glia were absent from spinal motor roots (Figure 6B,C,E). Treatment with DAPT at 46 hpf also resulted in a significant reduction in the number of MEP glia along motor roots (Figure 6B,D,E). Based on these studies, we hypothesize that MEP glia are generated from the same early spinal cord precursors that develop between 24 and 46 hpf and generate OPCs.

### MEP Glia Express *wif1*

Taken together, our data are consistent with the hypothesis that MEP glia are a novel peripheral glial cell population. Therefore, we sought to identify more selective markers to allow for more in-depth characterization. We started by using *in situ* hybridization to determine if genes that have previously been described as markers of BCCs and/or Schwann cells were expressed by MEP glia. Of the six BCC markers that we tested (*wif1*, *cdh7*, *krox20*, *sema6a*, *sema6d*, *sema6dl*) (Table 1), only one, *wnt inhibitory factor 1 (wif1)*, showed consistent expression at the MEP (Figure 6F) [9]. Prior to 48 hpf, we never detected *wif1* expression along the spinal motor nerve root, which is consistent with our previous imaging and fate mapping data describing the development of MEP glia (Figure 6F). However, we did detect expression in previously reported tissues, including the lateral line (Figure 6F). At 48 and 56 hpf, in a pattern that was consistent with the regular spacing of spinal motor nerves, we saw expression along motor root axons (Figure 6F). These temporal expression data are consistent with the hypothesis that *wif1* labels MEP glia.

**Table 1:** Molecular description of spinal motor root-associated glial cells.

Cell	*sox10*	*foxd3*	*olig2*	*wif1*	*cdh7*	*krox20*	*sema6a*	*sema6D*	*sema6dl*
OPC	**+**	**−**	**+**	**−**	**−**	**−**	**−**	**−**	**−**
Schwann cell	**+**	**+**	**−**	**−**	**−**	**+**	**−**	**−**	**−**
BCC	**+**	N/A^a^	**−**	**+**	**+**	**+**	**+**	**+**	**−**
MEP Glia	**+**	**+**	**+**	**+**	**−**	**−**	**−**	**−**	**−**

a Expression of this gene has not been directly tested in BCCs.

Comparison of molecular markers in all glial populations found at the MEP.

To further confirm that *wif1* labels MEP glia, we assayed *wif1* expression in embryos lacking these cells. As mentioned above, MEP glia are absent in DAPT-treated larvae, and consistent with the hypothesis that *wif1* labels MEP glia, we observed significantly reduced *wif1* staining at the motor root in these larvae (Figure 6H). As an independent confirmation, we also assayed *wif1* expression in genetic mutants that affect MEP glial development. In embryos harboring a mutation in the receptor tyrosine kinase *erbb3b*, there is a complete absence of *sox10* ^+^ glia along the spinal motor nerve [38]. Therefore, we hypothesized that MEP glia must be absent. To confirm this, we used the above described photoconversion experiment to demonstrate that MEP glia are absent in *Tg(sox10:eos);erbb3b* mutants (Figure S5A). When we assayed for *wif1* expression, we also observed a significant reduction of *wif1* ^+^ cells along the motor root in *erbb3b* mutants (Figure 6G). Based on the temporal and spatial expression of *wif1* and its absence in embryos that lack MEP glia, we propose that *wif1* is a promising marker for MEP glia.

In summary, we report that MEP glia express the spinal cord precursor marker *olig2*, the Schwann cell markers *sox10* and *foxd3*, and the BCC marker *wif1*. Taken together, these data are consistent with the hypothesis that the *sox10^+^* cells we observe exiting the spinal cord at the MEP are distinct from previously described OPCs, Schwann cells, and BCCs (Table 1).

### MEP Glial Derivatives Are a Permanent Glial Population

**Figure 7:**
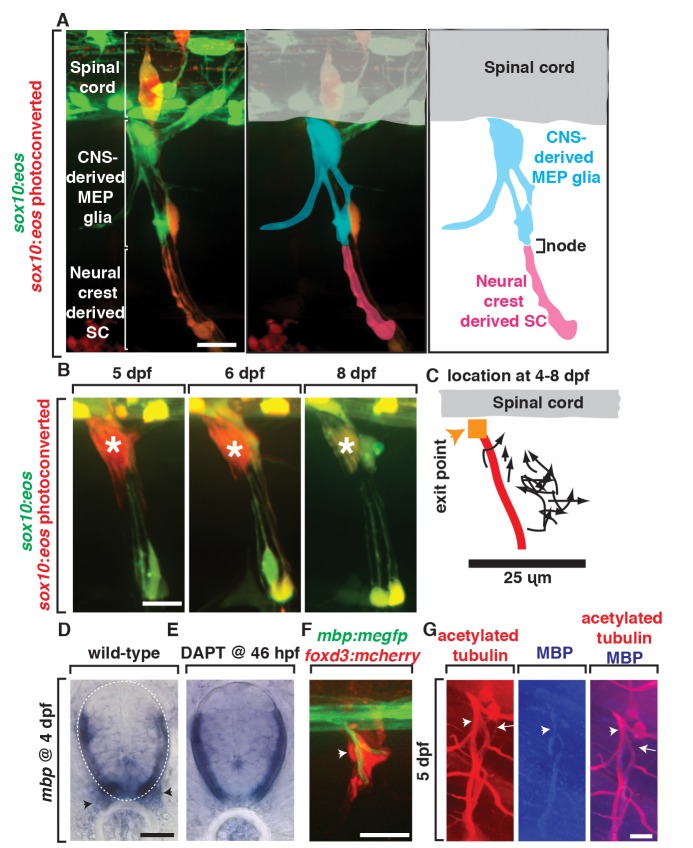
MEP glia and their descendents myelinate the spinal motor root. (A) In images from *Tg(sox10:eos)* animals exposed to UV at 48 hpf and imaged at 80 hpf, CNS-derived MEP glia ensheath the root and neural crest-derived Schwann cells (SCs) ensheath more distal portions of spinal nerves. CNS-derived MEP glia are overlayed with blue labeling and neural crest-derived SCs in pink. (B) In images of *Tg(sox10:eos)* embryos where single cells were photoconverted at 5 dpf and imaged on sequential days until 8 dpf, MEP glial derivatives remain ensheathed around spinal motor root axons. (C) Movement of single photoconverted MEP glia from 4–8 dpf. Orange box denotes the MEP, and red line denotes the motor axon. (D) *In situ* hybridization with *mbp* riboprobe showed *mbp^+^* staining (denoted by arrowhead) at the motor root at 4 dpf is absent in (E) DAPT-treated animals that lack MEP glia. (F) In a *Tg(mbp:megfp);Gt(foxd3:mcherry)* embryo at 4 dpf, *foxd3^+^* cells are located along a myelinated nerve (arrow). (G) In 5 dpf embryos stained with acetylated tubulin and MBP, MBP^+^ axons are present at the root where MEP glia reside. Scale bars, 25 µm.

Previously, glial cells along spinal motor root axons were thought to be a homogenous population of neural crest-derived glia [3],[39]. Our data, however, demonstrate that the motor root is ensheathed by glia that originate from the spinal cord. To characterize MEP glia and investigate the extent of their ensheathment along spinal motor root axons, we used fate-mapping to track MEP glial cell derivatives. To do this, we photoconverted *Tg(sox10:eos)* embryos at 48 hpf as described above and imaged at 80 hpf, once ensheathment of axonal segments had begun. Along the motor root, we observed only MEP glia and their derivatives (Figure 7A). However, further distally along these same axons, we observed neural crest-derived Schwann cells (Figure 7A). These two glial populations seamlessly ensheathed but were distinctly localized, separated only by a node-like gap (Figure 7A). Based on these data, we conclude that MEP glia and their derivatives ensheath only the spinal motor root.

We next asked if MEP glial derivatives were a transient population or if they remained along axons past development. To investigate this question, we photoconverted single MEP glial derivatives at the motor root and tracked the location of these single cells over several days. When individual cells were photoconverted at 4 dpf and imaged each day until 8 dpf, they remained in nearly the exact same location for the duration of the study (Figure 7B). We never observed cell death during this temporal window and did not detect any additional cell divisions (Figure 7). We also did not observe migration beyond what was required to ensheath the motor root (Figure 7C). Based on this evidence, we hypothesize that MEP glia generate cells that permanently ensheath the mature motor root.

**Figure 8:**
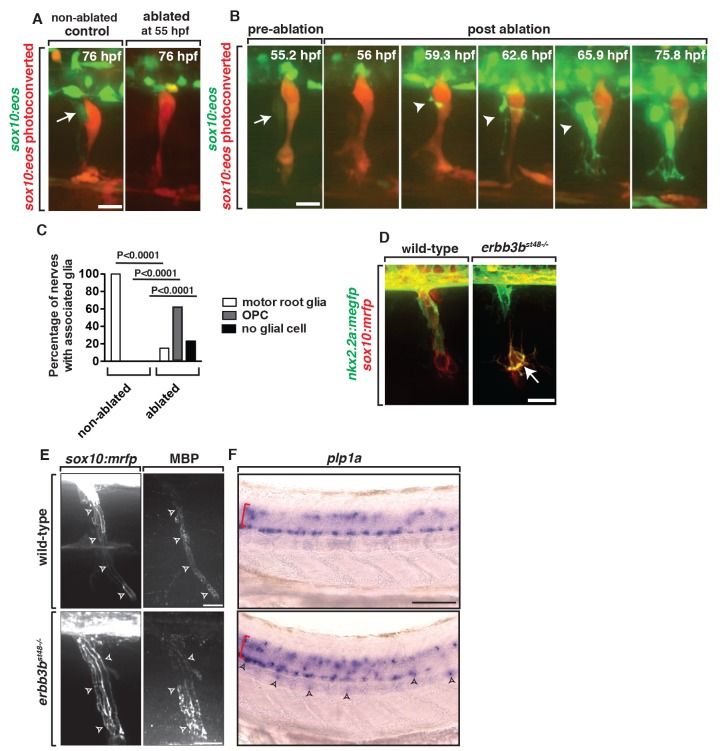
Ablation of MEP glia disrupts the MEP TZ. (A) In a control *Tg(sox10:eos)* embryo that was exposed to UV light at 48 hpf, motor (green) and sensory root (red) glial cells ensheath axons. When the CNS-derived MEP glial cell progenitor was ablated at 55 hpf and then imaged at 76 hpf, all *sox10^+^* motor root glial cells were absent. (B) Frames captured from a 16-h time-lapse video beginning at 56 hpf in a *Tg(sox10:eos)* embryo exposed to UV light at 48 hpf. Numbers in upper right corners denote stage of embryos. When the MEP glial cell progenitor (arrow) was ablated at 55 hpf, OPC processes (arrowhead) extended out of the spinal cord before OPC cell bodies exited. All images are lateral views of the motor and sensory root with dorsal to the top and anterior to the left. (C) Quantification of the data in panel B (*n* = 46 nerves). (D) In *Tg(nkx2.2a:mgfp);Tg(sox10:mrfp)*;*erbb3b^−/−^* embryos at 55 hpf, motor root glial cells are absent and OPCs (arrows) are in the PNS. (E) In *Tg(sox10:mrfp)* larva at 8 dpf stained with MBP, MBP^+^ cells were ensheathed around the root in wild-type and *erbb3* animals. (F) *In situ* hybridization of *plp1a* in wild-type and *erbb3b* mutant showed OPCs in the PNS in *erbb3b* mutants myelinate with CNS myelin. Scale bars, 25 µm.

We next asked if other peripheral glia were competent to ensheath the motor root in the absence of MEP glia. To do this we used a nitrogen-pulsed laser ablation system to specifically ablate these cells [40] after they exited the CNS, but before they divided, and assayed the presence of *sox10^+^* glial cells along motor axons later in development. When the *sox10^+^* cell at the MEP was ablated at 55 hpf and imaged at 76 hpf in *Tg(sox10:eos)* embryos, the majority of motor roots had no *sox10^+^* glia that had a morphology consistent with MEP glia (Figure 8A,C). Sensory glia adjacent to ablated MEPs, however, were unperturbed (Figure 8A). In the reciprocal experiment, when *sox10^+^* neural crest cells were ablated at 48 hpf, CNS-derived *sox10^+^* motor root glia were still present (Figure 3C). Based on these data, we conclude that MEP glia exclusively ensheath the motor root.

### MEP Glia Myelinate Spinal Motor Root Axons

Our above results demonstrate that the only *sox10* ^+^ glia that ensheath the root are MEP glia and their derivatives. To determine if they myelinate these spinal motor root axons, we first performed *in situ* hybridization on 4 dpf larvae with a riboprobe specific for *myelin basic protein* (*mbp*). In a pattern consistent with the regular spacing of spinal motor nerves, we detected *mbp* expression along motor roots at 4 dpf (Figure 7D). Because MEP glia are the only *sox10^+^* cell type found along the spinal motor root, we conclude that MEP glial derivatives myelinate spinal motor root axons. Consistent with this, we did not detect any *mbp* expression along spinal motor roots in DAPT-treated embryos that lack MEP glia (Figure 7E). To independently confirm these findings, we imaged *Gt(foxd3:mcherry);Tg(mbp:egfp)* embryos, which use *mbp* regulatory sequences to drive expression of membrane-tethered EGFP in all myelinating glia in both the CNS and PNS [41]. At 4 dpf, when *mbp^+^* membranes are first seen along spinal motor axons, they are tightly associated with *foxd3^+^* cells along spinal motor root axons (Figure 7F), consistent with the hypothesis that MEP glia myelinate motor axon roots. We then confirmed this cellular arrangement at 5 dpf with an antibody specific to zebrafish MBP (Figure 7G) [27]. Taken together, we conclude that MEP glia and their derivatives ensheath and myelinate spinal motor root axons.

### OPC Membrane Processes Are Repelled by MEP Glia

Our initial observation of OPC peripheral sampling at the MEP led us to hypothesize that PNS-located glia restrict OPCs from exiting the spinal cord. To test if MEP glia were the specific cells that served this repulsive function, we ablated them after they exited the spinal cord (∼55 hpf), but before they divided, and then time-lapse imaged embryos for 24 h. In these experiments, we used the photoconversion technique we describe above to specifically label the MEP glia for ablation with the nitrogen-pulsed laser. In embryos where MEP glia had been ablated, *sox10^+^* cells with morphology identical to OPCs exited the spinal cord (Figure 8B,C and Video S6). We confirmed that the *sox10^+^* cells that migrated into the PNS were OPCs by ablating the MEP glia in *Tg(olig2:dsred);Tg(sox10:eos)* embryos [21],[27]. These results support the hypothesis that the strict segregation of myelinating glia at the MEP TZ is achieved by contact-mediated inhibition between OPCs and MEP glia.

To complement these laser-ablation experiments, we investigated if OPCs exited the CNS in *erbb3b* mutant embryos [25],[38], which lack MEP glia (Figure 6G). We acknowledge that these mutants also lack neural crest-derived glial cells [25]. However, our laser-ablation results demonstrate that the neural crest-derived population of glial cells is not required to restrict OPCs to the spinal cord, as we never observed ectopic OPCs in the periphery when we specifically ablated neural crest-derived glia (Figure 3C). Consistent with our previous results, in *Tg(nkx2.2a:megfp);Tg(sox10:mrfp)*;*erbb3b* homozygous mutant embryos, we observed GFP^+^/RFP^+^ OPCs in the periphery (Figure 8D). Based on these two complementary approaches, we conclude that MEP glia are required to restrict OPCs to the spinal cord.

To further this analysis and investigate the long-term consequence of MEP glial ablation, we analyzed the motor root in *erbb3b* mutants later in larval development. First, we examined if ectopically located OPCs along the motor root myelinated peripheral motor axons. To do this, we used time-lapse imaging to characterize their behavior in *Tg(nkx2.2a:megfp);Tg(sox10:mrfp);erbb3b* homozygous mutants. These experiments showed that PNS-located OPCs transitioned from an exploratory behavior to a more static and less dynamic behavior by 75 hpf, consistent with their ensheathing activities (Figure S5B) [21]. To determine if they also myelinated motor axons, we labeled *erbb3b* homozygous mutants with an antibody to MBP. At 8 dpf, we observed significant MBP staining along motor root axons (Figure 8E). Because these mutants lack MEP glial derivatives and Schwann cells, any MBP staining along the motor nerve must be generated from ectopic OPCs [25],[38]. To determine if they maintain the ability to produce CNS myelin in the periphery, we assayed for expression of *plp1a*, a component of CNS myelin. At 4 dpf in *erbb3b* mutant larvae, we observed ectopically located *plp1a^+^* cells in the periphery via *in situ* hybridization (Figure 8F). We did not observe this staining in wild-type control animals (Figure 8F). We also did not observe any axonal debris or defasciculation at the motor root of these animals, which has previously been described [38]. We therefore propose that in the absence of MEP glia, OPCs ectopically myelinate the peripheral motor root with central myelin without causing any obvious axonal or neuronal phenotypes.

### OPC Processes Sample the PNS for Extended Periods When MEP Glia Are Absent

**Figure 9:**
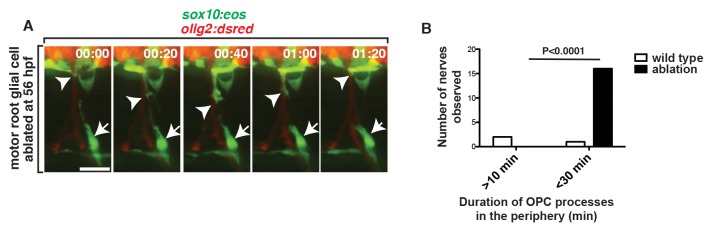
MEP glia restrict OPCs to the CNS. (A) Frames captured from a 24-h time-lapse video beginning at 56 hpf in a *Tg(sox10:eos);Tg(olig2:dsred)* embryo. Numbers in upper right corners denote time lapsed from the first frame of the figure. When the MEP glial cell progenitor was ablated at 56 hpf, OPCs (arrowhead) extended processes (arrowhead) into the periphery but retracted (60–80 min) after contacting MEP glia (arrow) at the horizontal myoseptum. (B) Quantification of data in panel A (16 OPC ectopic exit events were scored). Scale bar, 25 µm.

Our data demonstrate that MEP glia physically repel OPC processes and we hypothesized that this contact-mediated inhibition is what establishes the strict segregation of myelinating glia at the MEP TZ. If this hypothesis is correct, then we reasoned that OPCs would sample the periphery for longer periods if they did not encounter MEP glia at the TZ. To test this hypothesis, we time-lapse imaged embryos in which we ablated the MEP glial cell derivatives closest to the TZ and assayed the behavior of OPC processes that extended into the periphery. Along motor nerves where the CNS-derived *sox10^+^* glial cell closest to the MEP was ablated at 56 hpf, but its descendants further distal along on the nerve were intact, we observed OPC processes in the periphery for longer than 60 min (Figure 9A,B). These processes broadly surveyed the PNS and extended as far as the horizontal myoseptum (Figure 9A). We also noticed in ablated animals that OPCs frequently contacted neural crest-derived sensory glia (Movie S6). However, this contact did not result in retraction back into the spinal cord (Movie S6). Additionally, if the MEP glial derivatives prevented OPC migration by simply providing a physical barrier, then once OPC processes sampled the periphery in its absence, OPC cell bodies should migrate into the periphery. However, we observed OPC processes retracting back into the spinal cord only after they contacted MEP glial derivatives at the horizontal myoseptum (Figure 9A), suggesting that OPC peripheral migration is not solely inhibited by a physical barrier, but more likely repelled by a contact-mediated inhibition signal provided by CNS-derived MEP glial derivatives. Based on these data, we conclude that CNS-derived MEP glia and their descendants are essential for establishing and maintaining myelinating glial segregation at the MEP TZ through contact-mediated inhibition.

## Discussion

**Figure 10:**
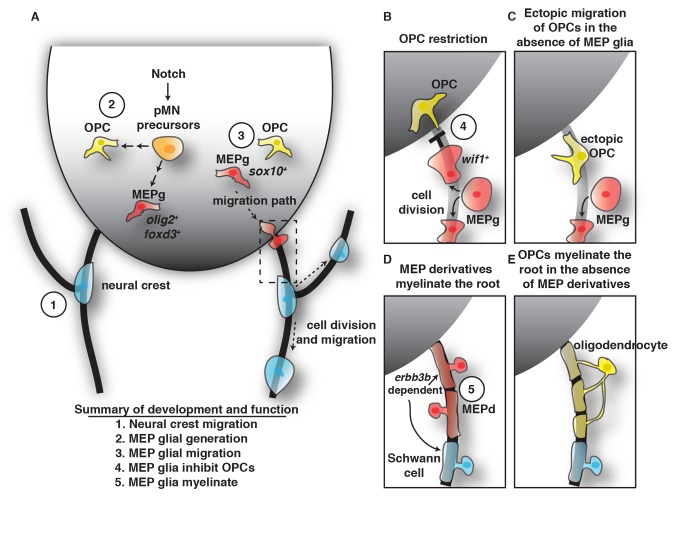
Summary of MEP glial development and function. (A) Schematic of a cross-section of the spinal cord summarizing the findings that pMN precursors (orange cells) generate OPCs (yellow cells) and *olig2*/*foxd3* expressing MEP glia (MEPg) (red cells). These precursors are dependent on Notch signaling. MEPg then initiate *sox10* expression, migrate to the MEP (dashed box), squeeze through the TZ, and reside along spinal motor root axons. (B–D) Inset of the MEP depicted as dashed box in (A). (B) OPCs are restricted from entering the PNS by *wif1^+^* MEPg. (C) In the absence of MEPg, OPCs ectopically migrate out of the spinal cord. (D) MEP glial derivatives (MEPd) myelinate the root and in their absence, and (E) oligodendrocytes myelinate motor root axons with central myelin. The association of MEPd and Schwann cells with the root is *erbb3b* dependent. Each developmental stage of MEPg is numerically labeled, showing MEPg (1) are independent of neural crest, (2) are generated from ventral spinal cord precursors, (3) migrate into the periphery via the MEP TZ, (4) inhibit OPC migration, and (5) myelinate the root.

During nervous system development, myelinating glia are arranged such that motor axons are myelinated by distinct populations of glia in the CNS versus PNS [3],[4],[20],[42]. This unique and precise organization of glia is essential for the rapid conduction of action potentials along motor axons and, under normal circumstances, is strictly maintained. However, the mechanism that establishes and maintains this arrangement is poorly understood. In this study, we demonstrate that OPC membrane processes sample the PNS prior to the onset of myelination and that they are actively restricted from migrating into the periphery via contact with a novel CNS-derived population of glia we call MEP glia. MEP glia are found specifically along spinal motor root axons; express *sox10*, *foxd3*, *olig2*, and *wif1*; and divide to selectively ensheath motor, but not sensory, root axons (Figure 10). When CNS-derived MEP glia are ablated, OPCs immediately exit the spinal cord and myelinate peripheral axon segments. From these data, we conclude that MEP glia are essential for segregating myelinating glia at the MEP TZ and that contact-mediated inhibition between central and peripheral glia is required to establish the MEP TZ (Figure 10).

In mammals, *sox10^+^* cells that associate with motor root axons are described as neural crest cells that give rise to Schwann cells and BCCs [3],[11],[39]. Because the cells we describe do not fit into the traditional description of Schwann cells, we feel it is inappropriate to classify them as such. BCCs in mammals are thought to restrict both neurons and glia at the MEP TZ [10],[11],[14]. Although an analogous cell type has not yet been identified in zebrafish, the MEP glial cells we describe are not like mammalian BCCs because they are not neural crest-derived, do not express most of the molecular markers of mammalian BCCs, and do not restrict motor neurons to the spinal cord [27]. Perineurial glia, the only other motor axon-associated peripheral glial cell type that has been described in zebrafish, is also distinct from MEP glia because: (1) their timing of development is distinct from MEP glia [6], (2) elimination of perineurial glia does not abolish MEP glia, and (3) the lack of perineurial glia does not result in OPCs along spinal motor root axons. Therefore, we conclude that the cell type we describe is not consistent with any other previously described spinal motor nerve root cell population.

### Do PNS Glial Cells Really Come from the Spinal Cord?

Although initially surprising, this feature has been observed in nonvertebrates like *Drosophila*, where ensheathing glia along motor axons originate inside the ventral nerve cord and migrate into the periphery [43]. Similarly, in zebrafish and mice, perineurial glia migrate from the floor plate to the MEP, where they pinch through the TZ and exit the spinal cord to ensheath motor nerves and ultimately form the mature perineurium [6],[44]. Our time-lapse imaging supports the hypothesis that MEP glia also originate within the spinal cord. Using multiple transgenic lines to label these cells and the border of the spinal cord, we show that *foxd3^+^/olig2^+^/sox10^+^* cells migrate through the MEP TZ and associate with spinal motor root axons. We acknowledge that our MO experiments do not distinguish between the two models in that these cells are generated from the *olig2^+^* domain versus that their development requires other cells that are impacted by perturbation of *olig2*. However, because the MEP glia migrate from a region close to the pMN and express *olig2*, we believe there is a strong possibility they are generated from pMN domain precursors. The absence of MEP glia in DAPT-treated animals further endorses this possibility. We believe this cell has been missed in previous studies because it expresses multiple neural crest markers (e.g., *sox10; foxd3*) and because mutants that disrupt neural crest association with the nerve also impact the development of this cell (e.g., *erbb3*, *erbb2*, *mont blanc;mother superior*) [25],[27]. Because of these previously published results, we considered the possibility that the cell was a misrouted neural crest cell but believe our experiments strongly rule out this possibility. First, the transgenes we use to label neural crest [e.g., *Tg(sox10:eos*), Gt(*foxd3:mcherry*)] have been well characterized to label all neural crest cells, and therefore, it is unlikely that MEP glia are nonlabeled neural crest cells that turn on *sox10^+^/foxd3^+^* in the spinal cord [26],[36]. A second possibility is that MEP glia are generated from a neural crest cell that migrates into the spinal cord as a *sox10^+^/foxd3^+^* cell, turns off this expression, and then reinitiates *sox10* and *foxd3* expression before it migrates out of the spinal cord. Given that the Eos protein perdures for longer than 24 h in our experiments, this possibility seems unlikely. In summary, we show that MEP glia express a cocktail of glial progenitor markers (e.g., *foxd3*, *sox10*, *olig2*, *wif1*) that have not previously been described in the same glial cell. We therefore named these glia based on their morphological and positional association with the MEP.

### Is There Evidence for MEP Glia in Other Vertebrates?

In elegant experiments in which the entire neural crest was removed from chick embryos, ensheathing cells still developed along spinal motor axons in chick [16]–[18]. These ensheathing cells were hypothesized to be derived from the spinal cord and remained at the motor root even in the absence of neural crest [16]–[18]. These results are consistent with our data that show MEP glia ensheath motor root axons even after neural crest ablation (Figure 3). In rodents, studies have described morphologically distinct glial cells at the MEP and dorsal root TZ, suggesting that more than one cell type may be present at the TZ [1],[15]. Additionally, in a rodent model of remyelination, Olig2*^+^* progenitors in the spinal cord can produce peripheral glial subtypes [45]. Although this remyelination study did not demonstrate that the Olig2-derived cells exit the spinal cord, their presence is consistent with the possibility that they may also exist during earlier stages of development. Future studies exploring this possibility are needed to definitively determine whether MEP glia are present in other vertebrates. However, given our data and the evidence previously described in chick, we hypothesize this is a strong possibility.

In vertebrates, glial cells at the spinal cord TZs are important to maintain CNS/PNS cellular segregation [10]. However, it is not clear how these cells communicate across the TZ given that they are largely segregated to their specific domains. We show here using *in vivo* time-lapse imaging in zebrafish that OPCs communicate with peripheral glial cells by extending thin, dynamic processes into the PNS, a phenomenon that would have been nearly impossible to observe in any other model organism. These OPC processes, however, retract when they contact MEP glia. We believe this heterotypic repulsion event between OPCs and MEP glia mediates the tight glial boundary at the MEP and is consistent with EM analysis of the myelin interphase at spinal cord TZs. Contact-mediated repulsion of glial membrane has been visualized in the CNS between two OPCs [21],[22] and this homotypic repulsion ensures that oligodendrocytes nonredundantly ensheath spinal cord axons [21]. We report here that repulsion can also occur between distinct classes of glia across the MEP TZ. The common origin of these cells and shared molecular characteristics may suggest a similar mechanism of repulsion is activated in both homotypic and heterotypic retraction and future studies investigating the nature of these interactions are required to understand the underlying molecular mechanisms of these two contact-mediated inhibition types. Together our results indicate that glial cells found on the peripheral side of the MEP originate in the CNS, ensheath spinal motor root axons, and are essential to maintain the basic architecture of the nervous system by restricting OPCs to the spinal cord.

## Materials and Methods

### Fish Husbandry

All animal studies were approved by The University of Virginia Institutional Animal Care and Use Committee (Protocol No. 3782). Zebrafish embryos and larvae were anesthetized using Tricaine, also known as Mesab or MS-222 (3-aminobenzoic acid ester). Euthanasia used an overdose of Tricaine. The following zebrafish strains were used in this study: AB*, *Tg(neurod:egfp)^nl1^* [28], *Tg(olig2:dsred2)^vu19^* [6], *Tg(sox10(7.2):megfp)*, *Tg(sox10(7.2):mrfp)^vu234^* [6],[21], *Tg(nkx2.2a:megfp)^vu17^* [21], *Tg(sox10(4.9):eos)*, *Tg(sox10(4.9):nls-eos)* [26], *Tg(Xla.Tubb:DsRed)* [46], *Gt(foxd3-mcherry)^ct110aR^* [36], *Tg(neurog1:gfp)^w61^*, *Tg(gfap:egfp)* [47], *Tg(mbp:egfp-caax)* [41], and *erbb2b^st61^* and *erbb3b^st48^* [38]. Abbreviations used for each line are denoted in Table S1. Embryos were produced by pairwise matings, raised at 28.5°C in egg water, and staged by hpf and dpf [48]. Embryos used for immunohistochemistry and live imaging were treated with 0.004% phenylthiourea (PTU) in egg water to reduce pigmentation. All lines used were stable, germline transgenics.

### In Vivo Imaging

All animals for imaging were manually dechorinated at 24 hpf and treated with PTU as described above. At specified stages, embryos were anesthetized with 3-aminobenzoic acid ester (Tricaine), immersed in 0.8% low-melting point agarose, and mounted on their sides in glass-bottomed 35 mm Petri dishes (Electron Microscopy Sciences). Images were captured with a 25× multi-immersion objection (numerical aperture = 0.8), a 40× water objective (numerical aperture = 1.1), or a 63× water objective (numerical aperture = 1.2) mounted on a motorized Zeiss AxioObserver ZI microscope equipped with a Quorum WaveFX-XI spinning disc confocal system (Quorum Technologies Inc.). Z-stacks were collected that covered the span of the nerves. Three-dimensional and single-plane datasets were processed in MetaMorph. Supporting videos were annotated and created using ImageJ. Cell tracking annotation was done with the MTrackJ plugin. Photoshop was used to enhance brightness and contrast of images.

### Eos Photoconversion

Animals were treated with PTU and mounted for *in vivo* imaging as described above. To photoconvert all *Tg(sox10:eos)* cells, embryos were exposed to UV light through a DAPI filter for 20–30 s using a 20× objective. For single-cell photoconversion, a MicroPoint laser with LD390/Stillbeme 420 (404 nm) dye was used.

### Chemical Treatments and Morpholino Injections

Embryos were treated with 50 µM CA diluted in egg water at 8 hpf. Zebrafish embryos were treated with 100 µM DAPT [565784; N-(3,5–difluorphenyl-L-alanyl-2-phenyl glycine-1,1-dimethethyl) ester; EMD Chemicals] diluted in 1% DMSO in PTU egg water at the designated time. Control embryos were placed in 1% DMSO in egg water. *nkx2.2a* MO and *olig2* MO were diluted from a stock solution of 3 mM, diluted in 2× injection buffer to create a working concentration of 0.5 mM, and injected into single-cell embryos. All MO-injected and drug-treated animals were mounted as discussed above for live imaging. Fifty nerves in five different embryos were scored for each condition of CA, wild-type, and *nkx2.2a* MO-injected and 88 nerves in 10 different animals were scored in *olig2* MO-injected embryos.

### Immunohistochemistry

Animals were fixed with AB fix [4% Paraformaldehyde and 1×PBST (1% TritonX)] for 3 h at 23°C and then washed in 1×PBST (5% TritonX), ddH20Tx (5% TritonX), and acetone for 5 min each at 23°C and then an additional acetone wash at −20°C. 1×PBST (5% TritonX) with 5% goat serum was used to block for at least an hour. Animals were incubated in primary antibody overnight at 4°C. The primary antibodies used in this study include the following: Sox10, 1∶5,000 [49]; Acetylated Tubulin, 1∶10,000 (Sigma); HuC, 1∶100 (Invitrogen); MBP, 1∶250 [27]. Animals were washed extensively with 1×PBSTx before the secondary antibody was added. These antibodies include Alexa antibodies (1∶600): goat anti-rabbit 568, goat anti-mouse 568, goat anti-rabbit 647, and goat anti-mouse 647. After extensive washes, animals were stored in 50% glycerol/50% 1×PBS until imaged and mounted under a bridged coverslip.

### *In Situ* RNA Hybridization

Larvae were fixed in 4% paraformaldehyde for 24 h, stored in 100% methanol at −20°C, and processed for *in situ* RNA hybridization. Plasmids were linearized with appropriate restriction enzymes and cRNA preparation was performed using Roche DIG labeling reagents and the appropriate RNA polymerase. After *in situ* hybridization, embryos were either imaged whole mount or embedded in 1.5% agar/30% sucrose and frozen in 2-methylbutane chilled by immersion in liquid nitrogen. Transverse sections (20 µm) were collected on microscope slides using a cryostat microtome and covered with 75% glycerol. Images were obtained using a Zeiss AxioCam CCD camera mounted on a Zeiss AxioObserver Z1 microscope equipped with Zeiss AxioVision software. All images were imported into Adobe Photoshop. Adjustments were limited to levels, contrast, color match settings, and cropping.

### Cell Ablation

Single-cell ablations were done with a MicroPoint Laser in conjunction with a coumarin dye (440 nm) with a 63× objective. Once a pre-ablation image was captured, a region of interest (ROI) was created based off of the merged-color image to specifically ablate cells. As a control for the ablation, regions surrounding the experimental ROI were ablated, including anterior to the nerve, posterior to the nerve, and dorsal to the MEP. In all control paradigms, OPCs did not exit the spinal cord.

### Data Quantification and Statistical Analysis

To count cells in experimental and control larvae, composite Z image stacks were compiled using Metamorph software. Cell counts were taken from lateral views of the spinal cord. Individual Z images were sequentially observed and cells counted within the entire Z stack. All graphically presented data represent the mean of the analyzed data. Statistical analyses were performed with GraphPad Prism software. The level of significance was determined by using a Chi-squared two-tailed test using a confidence interval of 95%.

## Supporting Information

Figure S1 OPCs contact peripheral spinal motor root glia. Frame captured from a 14-h time-lapse movie beginning at 58 hpf in a *Tg(sox10:eos);Tg(olig2:dsred)* embryo exposed to UV light at 48 hpf. At approximately 68 hpf, motor root glial (MG) cells (green, arrow) and OPCs (yellow/green, arrowhead) can be seen. Orthogonal view of the YZ plane shows OPC process (arrowhead) contacting a motor root glial cell (arrow). Traced schematic of the YZ plane below.
(TIF)

Figure S2 Motor root *sox10^+^* cells originate within the CNS. (A) Frames of single optical planes captured from a 24-h time-lapse movie beginning at 48 hpf in a *Tg(sox10:eos);Tg(ntb:dsred)* embryo. Numbers in upper right corners denote time lapsed from the first frame of the figure. At approximately 54 hpf, a *sox10^+^* cell (arrow) migrated from a dorsal location in the CNS (00:00), exited the spinal cord at the MEP, and associated with motor nerve axons. (B) The path of migration of the motor root glial cell progenitor depicted above. The traced schematic below represents this migration and subsequent cell divisions. Scale bars, 25 µm.
(TIF)

Figure S3 Photoconversion technique used to distinguish between motor and sensory glia. (A) Schematic of experimental design demonstrates that embryos were exposed to UV light at 48 hpf to photoconvert all neural crest *sox10^+^* cells from green to red. Cells that turn on Eos expression after 48 hpf are labeled with unconverted Eos protein (green). nc, neural crest cell; mg, motor glia. (B) In *Tg(sox10:eos)* embryos exposed to UV light at 48 hpf, fixed at 80 hpf, and labeled with antibodies specific to acetylated tubulin and HuC, photoconverted cells (red, arrowhead) were associated with sensory axons (blue), while unconverted cells (green, arrow) ensheathed motor axons (blue, arrow). Scale bar, 15 µm.
(TIF)

Figure S4 Two physically distinct glial populations are present along motor and sensory axons. Confocal images of a *Tg(sox10:megfp);Tg(olig2:dsred)* embryo at 54 (A) and 72 (B) hpf show *sox10^+^* motor root glial cells (arrow) ensheathing motor axons (red) and *olig2^−^* sensory axons (arrowhead). (C) Confocal image of a *Tg(sox10:mrfp);Tg(neurod:gfp)* embryo at 54 hpf showing *sox10^+^* cells along sensory axons (green, arrowhead) and *neurod^−^* motor axons (arrow). (D) In a *Tg(neurod:gfp);Tg(sox10:mrfp)* larva at 8 dpf, two distinct *sox10^+^* fascicles of sensory (arrowhead) and motor (arrow) axons were seen. Scale bars, 25 µm.
(TIF)

Figure S5 MEP glia are absent in *erbb3b* mutant larvae. (A) In a *Tg(sox10:eos);erbb3b* mutant embryo exposed to UV light at 48 hpf and imaged at 72 hpf, MEP glia were absent and OPCs (arrowhead) were observed in the periphery. (B) Frames captured from a 15-h time-lapse movie beginning at 54 hpf in a *Tg(nkx2.2a:megfp);Tg(sox10:eos);erbb3b^−/−^* embryo. Numbers in lower left corners denote stage of embryo. Arrowheads denote OPCs that are ensheathing motor axons. Scale bar, 25 µm.
(TIF)

Table S1 Descriptions and abbreviations of transgenic lines used in this study. All lines used were stable, germline transgenics. Cell types listed for each transgene are only those pertinent to this study.
(DOCX)

Data S1 Excel spreadsheet containing, in separate sheets, the underlying numerical data and statistical analysis for Figures 3B, 4C, 6E, 8C, and 9B.
(XLSX)

Video S1 OPC processes sample the periphery during normal development. Excerpt from a 14-h time-lapse of a *Tg(sox10:eos)* embryo from 58 to 72 hpf. *sox10^+^* cells (cell in the CNS labeled with black dot) extended dynamic processes into the periphery that contacted motor root glial cells (cell in periphery labeled with grey dot). After contact, OPCs remained in the CNS. At 90 min, the Supporting Video was frozen to display the OPC process in the periphery. Video on the left is annotated. Video on right is unannotated. Images were taken every 2.5 min, and the video runs at 10 fps.
(MOV)

Video S2 *sox10* ^+^ glia exit the spinal cord after neural crest migration ceases. Excerpts from a 24-h time-lapse of a *Tg(sox10:eos)* embryo that was imaged laterally (left) and turned digitally 90 degrees (right) to visualize an optical cross-section of the spinal cord. A *sox10^+^* cell migrated ventrally from the spinal cord, pinched at the MEP as it exited, and remained outside of the spinal cord. Video on the left is annotated with the dots marking the MEP glia cell. Video on the right is unannotated. Images were taken every 5 min, and the video runs at 10 fps.
(MOV)

Video S3 Motor root glial cells have dynamic processes. Excerpt from an 18-h time-lapse of a *Tg(sox10:eos)* embryo that was exposed to UV light at 48 hpf and imaged from 54 to 72 hpf. Thin dynamic processes extended from the motor root glial cell throughout development. By 72 hpf, both dorsal and ventral motor roots were ensheathed by unconverted *sox10* ^+^ cells. Video on the left is annotated with the white dot marking the cell body and the white open circle labeling the dynamic filopodium-like processes. Images were taken every 5 min, and the video runs at 3 fps.
(MOV)

Video S4 *olig2^+^* cells migrate from the spinal cord and ensheath motor root axons. Excerpts from a 24-h time-lapse of a *Tg(olig2:dsred)* embryo that was imaged laterally. *olig2* ^+^ cells migrated ventrally from the spinal cord, pinched at the MEP, and then ensheathed the motor root. Video on the left is unannotated, and the video on the right is annotated by color dots. Each cell division is annotated with a different color dot. Images were taken every 15 min, and the video runs at 10 fps.
(MOV)

Video S5 MEP glia express *foxd3* during migration out of the spinal cord. Excerpt from a 24-h time-lapse of a *Gt(foxd3:mcherry);Tg(gfap:egfp)* embryo imaged at 48 hpf. *foxd3^+^* cells start in the spinal cord, migrate through the *gfap^+^* endfeet at the MEP, and then ensheath the area where the motor axon is located. Video on the left is a lateral view with annotation that marks the *foxd3^+^* MEP glia with a blue dot. The middle video has the same time points but turned 90 degrees to visualize a cross-section through the spinal cord. Note the endfeet mark the edge of the spinal cord. The right video is the *Gt(foxd3:mcherry)* channel to show an unannotated view of MEP glial migration and development. Images were taken every 10 min, and the video runs at 10 fps.
(MOV)

Video S6 Motor root glial cells gate the MEP TZ. Excerpt from a 16-h time-lapse of a *Tg(sox10:eos)* embryo exposed to UV at 48 hpf and imaged from 56 to 72 hpf. After motor root glial cell progenitor ablation at 52 hpf, OPCs extended processes into the periphery and exited the CNS. Arrows denote the motor root glial cells before ablation. Left video is annotated with colored dots labeling OPC cells that will eventually exit and the open circle marking the cell processes of OPCs before the cell body exited the spinal cord. Images were taken every 10 min, and the video runs at 3 fps.
(MOV)

## Abbreviations

BCC: boundary cap cell
CNS: central nervous system
DPF: days postfertilization
DRG: dorsal root ganglia
HPF: hours postfertilization
MEP: motor exit point
MEPd: motor exit point derivative
MEPg: motor exit point glia
MG: motor glia
NC: neural crest
OPCs: oligodendrocyte progenitor cells
PNS: peripheral nervous system
TZ: transition zone
