# Supplementary figures and images for: Contact-Mediated Inhibition Between Oligodendrocyte Progenitor Cells and Motor Exit Point Glia Establishes the Spinal Cord Transition Zone
Source: PLoS Biol. 2014 Sep 30;12(9):e1001961. doi: 10.1371/journal.pbio.1001961 (PMC4181976; doi:10.1371/journal.pbio.1001961)

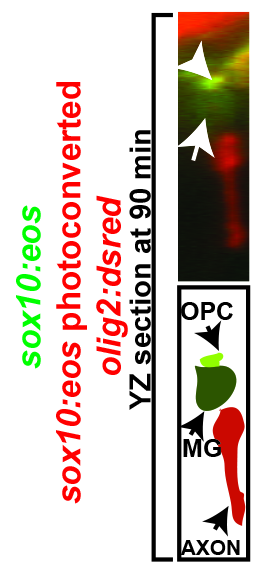

Supplement: Figure S1 — OPCs contact peripheral spinal motor root glia. Frame captured from a 14-h time-lapse movie beginning at 58 hpf in a Tg(sox10:eos);Tg(olig2:dsred) embryo exposed to UV light at 48 hpf. At approximately 68 hpf, motor root glial (MG) cells (green, arrow) and OPCs (yellow/green, arrowhead) can be seen. Orthogonal view of the YZ plane shows OPC process (arrowhead) contacting a motor root glial cell (arrow). Traced schematic of the YZ plane below. (TIF) [file pbio.1001961.s001.tif]

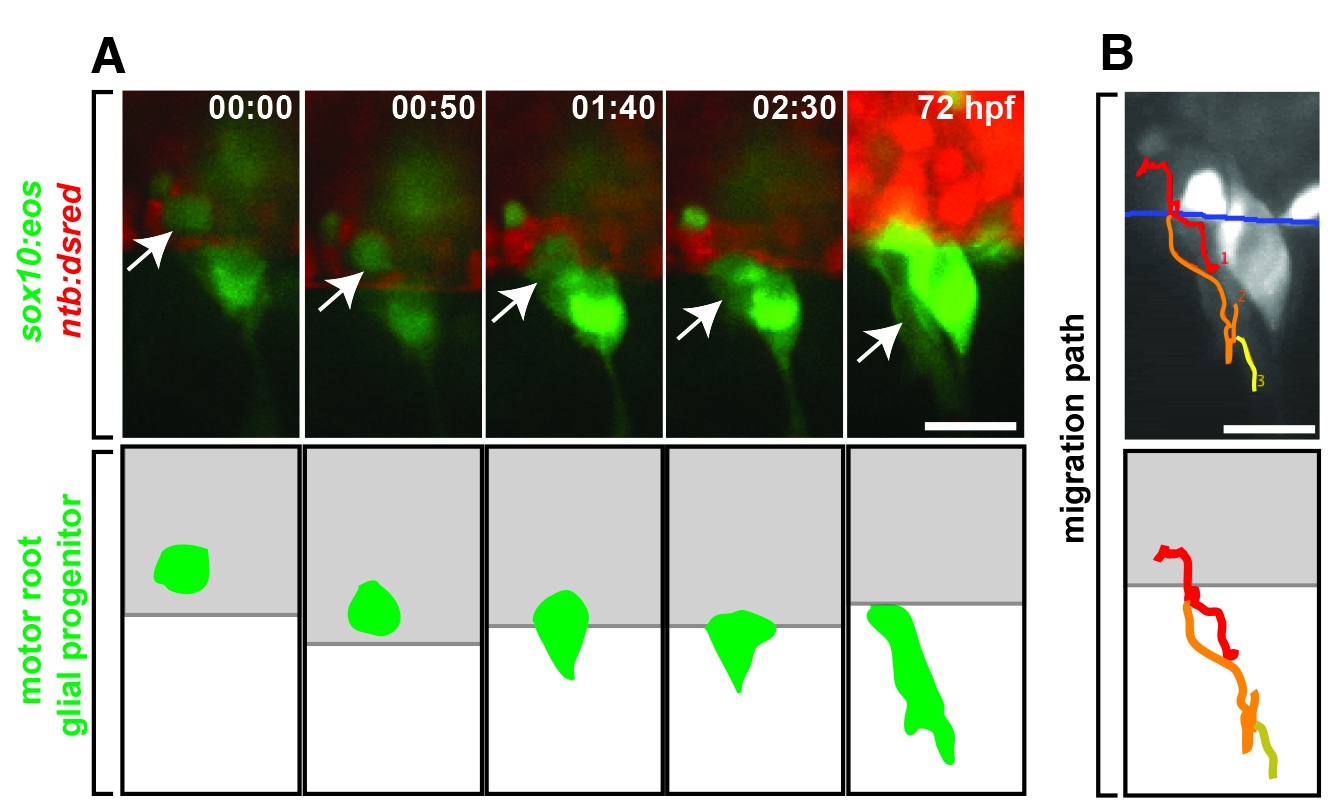

Supplement: Figure S2 — Motor root sox10+ cells originate within the CNS. (A) Frames of single optical planes captured from a 24-h time-lapse movie beginning at 48 hpf in a Tg(sox10:eos);Tg(ntb:dsred) embryo. Numbers in upper right corners denote time lapsed from the first frame of the figure. At approximately 54 hpf, a sox10+ cell (arrow) migrated from a dorsal location in the CNS (00:00), exited the spinal cord at the MEP, and associated with motor nerve axons. (B) The path of migration of the motor root glial cell progenitor depicted above. The traced schematic below represents this migration and subsequent cell divisions. Scale bars, 25 µm. (TIF) [file pbio.1001961.s002.tif]

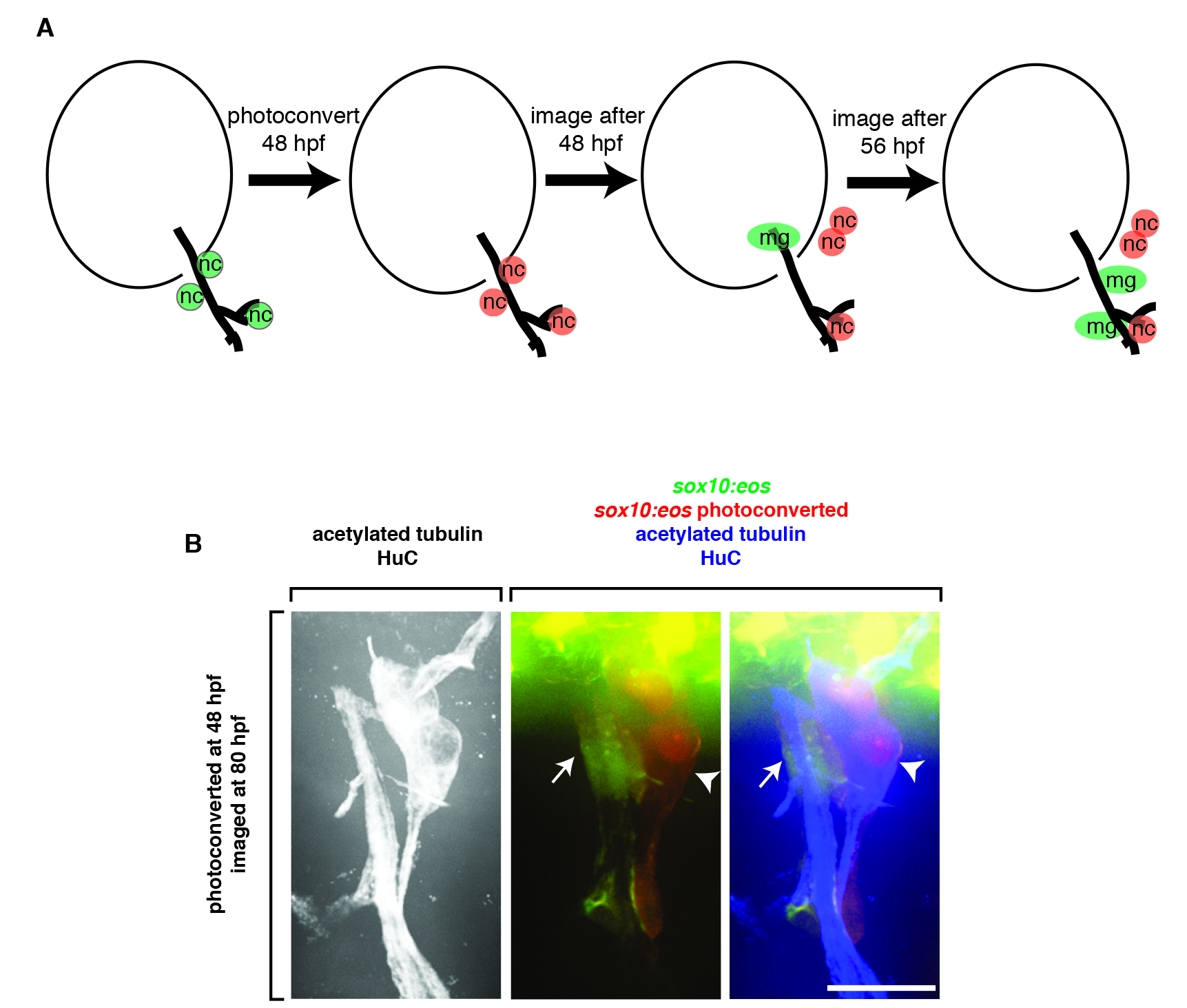

Supplement: Figure S3 — Photoconversion technique used to distinguish between motor and sensory glia. (A) Schematic of experimental design demonstrates that embryos were exposed to UV light at 48 hpf to photoconvert all neural crest sox10+ cells from green to red. Cells that turn on Eos expression after 48 hpf are labeled with unconverted Eos protein (green). nc, neural crest cell; mg, motor glia. (B) In Tg(sox10:eos) embryos exposed to UV light at 48 hpf, fixed at 80 hpf, and labeled with antibodies specific to acetylated tubulin and HuC, photoconverted cells (red, arrowhead) were associated with sensory axons (blue), while unconverted cells (green, arrow) ensheathed motor axons (blue, arrow). Scale bar, 15 µm. (TIF) [file pbio.1001961.s003.tif]

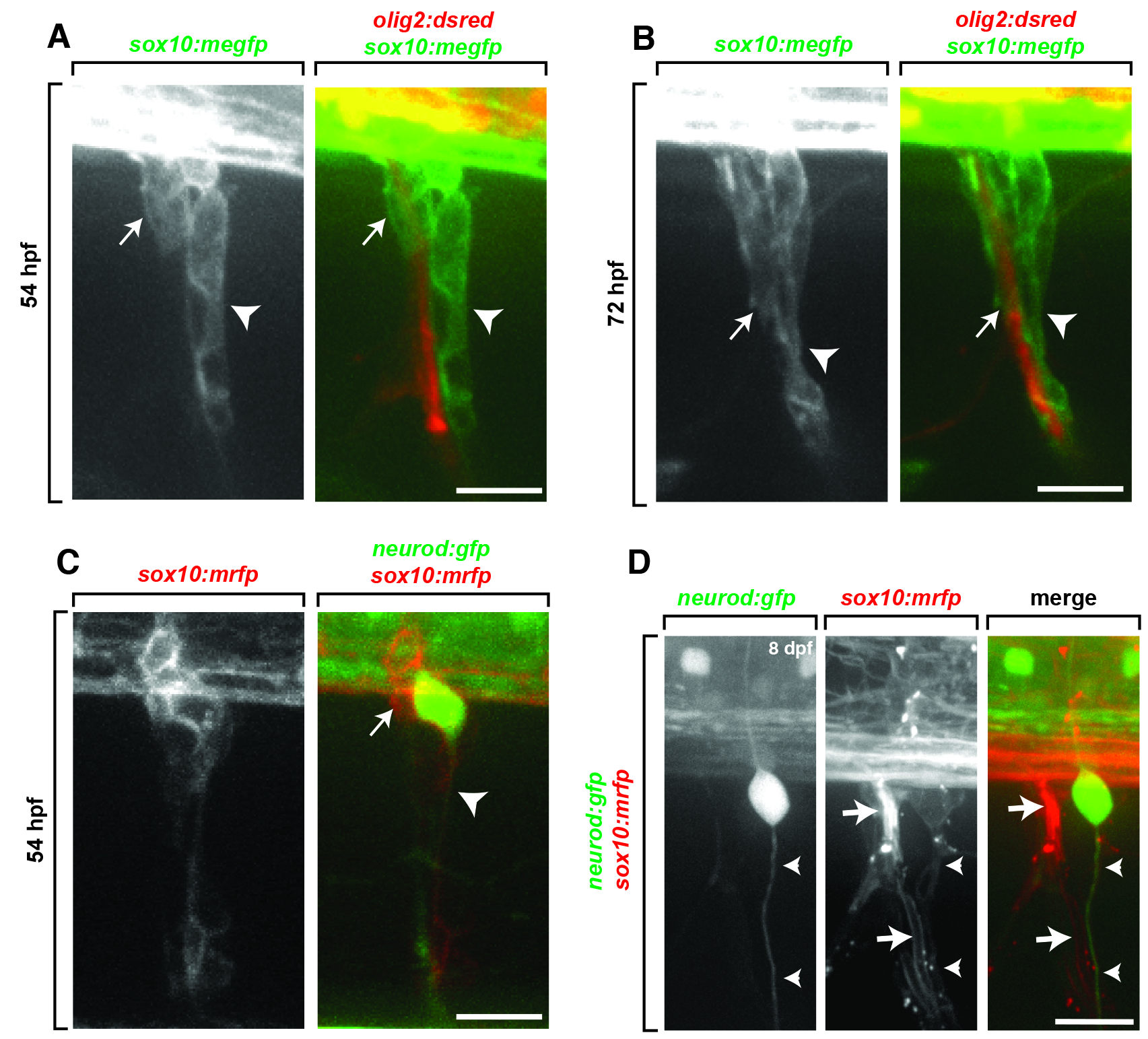

Supplement: Figure S4 — Two physically distinct glial populations are present along motor and sensory axons. Confocal images of a Tg(sox10:megfp);Tg(olig2:dsred) embryo at 54 (A) and 72 (B) hpf show sox10+ motor root glial cells (arrow) ensheathing motor axons (red) and olig2− sensory axons (arrowhead). (C) Confocal image of a Tg(sox10:mrfp);Tg(neurod:gfp) embryo at 54 hpf showing sox10+ cells along sensory axons (green, arrowhead) and neurod− motor axons (arrow). (D) In a Tg(neurod:gfp);Tg(sox10:mrfp) larva at 8 dpf, two distinct sox10+ fascicles of sensory (arrowhead) and motor (arrow) axons were seen. Scale bars, 25 µm. (TIF) [file pbio.1001961.s004.tif]

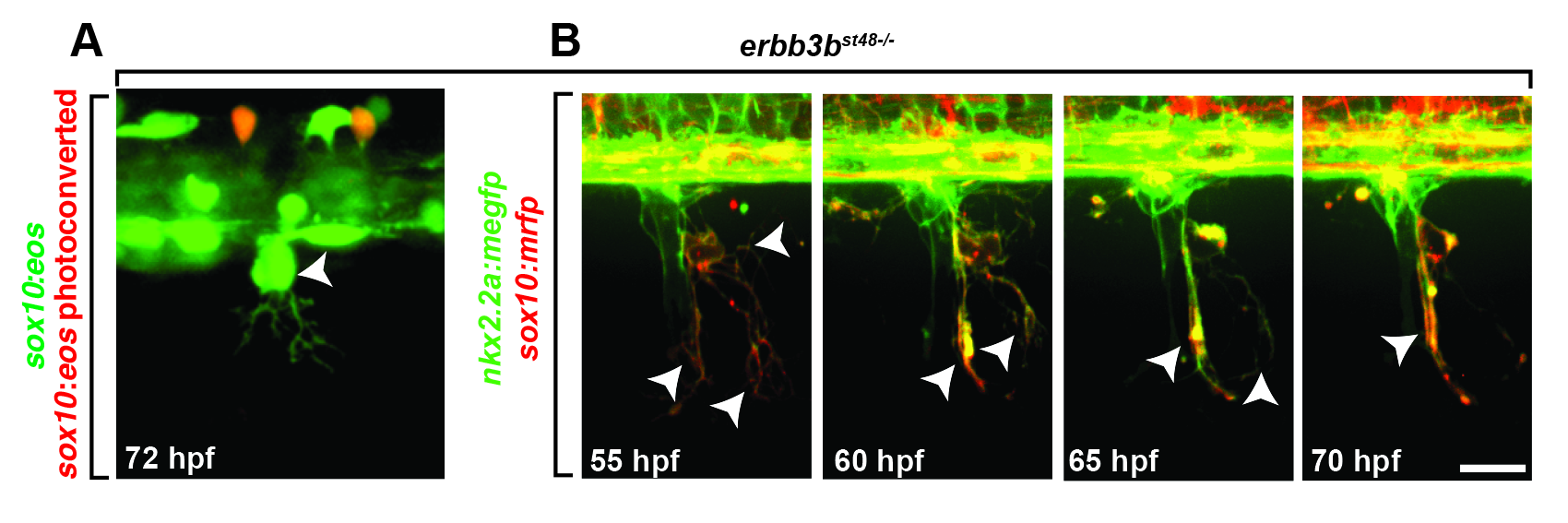

Supplement: Figure S5 — MEP glia are absent in erbb3b mutant larvae. (A) In a Tg(sox10:eos);erbb3b mutant embryo exposed to UV light at 48 hpf and imaged at 72 hpf, MEP glia were absent and OPCs (arrowhead) were observed in the periphery. (B) Frames captured from a 15-h time-lapse movie beginning at 54 hpf in a Tg(nkx2.2a:megfp);Tg(sox10:eos);erbb3b−/− embryo. Numbers in lower left corners denote stage of embryo. Arrowheads denote OPCs that are ensheathing motor axons. Scale bar, 25 µm. (TIF) [file pbio.1001961.s005.tif]
