# Supplementary material for: Contact-Mediated Inhibition Between Oligodendrocyte Progenitor Cells and Motor Exit Point Glia Establishes the Spinal Cord Transition Zone
Source: PLoS Biol. 2014 Sep 30;12(9):e1001961. doi: 10.1371/journal.pbio.1001961 (PMC4181976; doi:10.1371/journal.pbio.1001961)
Supplement: Table S1 — Descriptions and abbreviations of transgenic lines used in this study. All lines used were stable, germline transgenics. Cell types listed for each transgene are only those pertinent to this study. (DOCX) [file pbio.1001961.s006.docx]

| Transgene Name | Transgene abbreviation | Cell Labeled | Transgene action |
| --- | --- | --- | --- |
| *Tg(sox10(4.9):nls-eos)* | *Tg(sox10:nls-eos)* | Neural crest, Schwann cells and OPCs | Nuclear localized Eos expression in *sox10*^+^ cells |
| *Tg(sox10(4.9):eos)* | *Tg(sox10:eos)* | Neural crest, Schwann cells and OPCs | Eos expression in *sox10*^+^ cells |
| *Tg (sox10(7.2):mrfp)* | *Tg(sox10:mrfp)* | Neural crest, Schwann cells and OPCs | Membrane RFP expression in *sox10*^+^ cells |
| *Tg (sox10(7.2):megfp)* | *Tg(sox10:megfp)* | Neural crest, Schwann cells and OPCs | Membrane eGFP expression in *sox10*^+^ cells |
| *Tg(neurod:egfp)* | *Tg(neurod:gfp)* | DRG neurons/axons | eGFP expression in *neurod*^+^ cells |
| *Tg(olig2:dsred2)* | *Tg(olig2:dsred)* | Motor neurons/axons and OPCs | DsRed2 expression in *olig2*^+^ cells |
| *Tg(nkx2.2a:megfp)* | *Tg(nkx2.2a:megfp)* | Floorplate, OPCs and perineurial glia | Membrane eGFP expression in *nkx2.2a*^+^ cells |
| *Tg(Xla.Tubb:dsred)* | *Tg(nbt:dsred)* | All neurons | DsRed expression in all neurons |
| *Gt(foxd3-mcherry)* | *Gt(foxd3-mcherry)* | Neural crest, Schwann cells, DRG | Cherry expression in *foxd3*^+^ cells |
| *Tg(gfap:egfp)* | *Tg(gfap:egfp)* | Radial glia | eGFP expression in *gfap^+^* cells |
| *Tg(mbp:egfp-caax)* | *Tg(mbp:egfp)* | Myelinating glia | Membrane eGFP expression in all myelinating glia |
